# Caliber of Intracranial Arteries as a Marker for Cerebral Small Vessel Disease

**DOI:** 10.3389/fneur.2020.558858

**Published:** 2020-09-24

**Authors:** Zhaoyao Chen, Hui Li, Minghua Wu, Cheng Chang, Xinying Fan, Xinfeng Liu, Gelin Xu

**Affiliations:** ^1^Department of Neurology, The Affiliated Hospital of Nanjing University of Chinese Medicine, Nanjing, China; ^2^Department of Neurology, Jinling Hospital, Medical College of Nanjing University, Nanjing, China

**Keywords:** cerebral small vessel disease, white matter hyperintensity, lacuna infarct, enlarged perivascular spaces, brain arterial remodeling

## Abstract

**Background:** The dilation of intracranial large arteries caliber, may transfer more hemodynamic burden to the downstream brain capillaries, which, in the long run, results in cerebral small vessel disease (CSVD). This study aimed to investigate the relationship between intracranial artery calibers and small vessel disease.

**Methods:** Patients with first-ever ischemic stroke of lacunar infarction subtype were enrolled via Nanjing Stroke Registry Program. An intracranial arterial *Z*-score, named the brain arterial remodeling (BAR) score, was calculated by averaging the calibers of the seven main intracranial arteries. Among the enrolled patients, those with a BAR score < −1 SD were deemed to have small intracranial artery calibers; those with a BAR score >1 SD were deemed to have large intracranial artery calibers and those with a between BAR score were deemed to have normal intracranial artery calibers. Imaging markers of CSVD, including lacuna, white matter hyperintensity (WMH), enlarged perivascular spaces (EPVS) and cerebral microbleeds (CMBs) were rated and then summed to obtain a total CSVD score.

**Results:** A total of 312 patients were involved in this study, patients with BAR score >1 SD were older (*P* = 0.039), and more prone to having a history of myocardial infarction (*P* = 0.033). The Spearman's rank correlation coefficient between the BAR score and total CSVD score is 0.320 (*P* < 0.001). Binary logistic regression found that BAR score >1 SD was correlated with lacuna (OR = 1.987; 95% CI, 1.037–3.807; *P* = 0.039); severe WMH (OR = 1.994; 95% CI, 1.003–3.964; *P* = 0.049); severe EPVS (OR = 2.544; 95% CI, 1.299–4.983; *P* = 0.006) and CSVD (OR = 2.997; 95% CI 1.182–7.599; *P* = 0.021). Ordinal logistic regression analysis found that age (OR = 1.028; 95% CI, 1.007–1.049; *P* = 0.009), hypertension (OR = 3.514; 95% CI, 2.114–5.769; *P* < 0.001) and BAR score >1 SD (OR = 2.418; 95% CI, 1.350–4.330; *P* = 0.003) were correlated with the total CSVD score.

**Conclusions:** Patients with large intracranial arterial calibers may have heavier CSVD burden. The mechanisms of this association warrant further study.

## Introduction

A previous study suggested extreme brain arterial diameters correlated with vascular death, myocardial infarction, and any vascular event (1). Our previous study also found that a dilated basilar artery correlated with stroke recurrence (2). As the intermediary arteries connect extracranial arteries and cerebral small vessels, intracranial large arteries dampen the systematic pressure and pulsatility that are transmitted to brain capillaries (3). The dilation of arterial caliber may reduce the capability of physiological cerebral autoregulation and cause end-organ damage, such as cerebral small vessel disease (CSVD).

A previous study found that a larger carotid lumen diameter (but not common carotid artery intima-media thickness) was associated with a higher prevalence of lacunar infarcts (LI) (4). In addition, carotid stiffness was associated with increased white matter hyperintensity (WMH) volume which was independent of carotid plaque (4). Middle cerebral artery (MCA) diameter, as a surrogate of stiffness, is associated with anterior enlarged perivascular spaces (EPVS), and the association is the strongest among individuals with dilated brain arteries (5). Additionally, extreme intracranial arterial enlargement, in some cases called intracranial arterial dolichoectasia, also correlated with lacuna, WMH, EPVS (6), and cerebral microbleeds (CMBs) (7). These findings suggested that intermediary arteries could modify the association of extracranial pressure and CSVD, as observed in LI, WMH, and EPVS.

However, the intracranial arterial caliber and its relationship with the total burden of CSVD remain unknown. We hypothesized that the brain capillaries of patients with large intracranial arterial calibers may be exposed to a more severe hemodynamic burden and may suffer a heavier burden of CSVD.

## Method

### Patients

Consecutive patients with first-ever lacuna stroke proven by magnetic resonance imaging (MRI) were retrieved from Nanjing Stroke Registry (8) from September 1, 2015, through August 31, 2016. We excluded patients with symptomatic large artery stenosis (≥50%) and patients with possible cardioembolic sources such as atrial fibrillation, valvular heart disease or cardiac valve replacement. We also excluded patients if they had no brain MRI source images which were used for the measurement of intracranial artery diameters.

### Vascular Risk Factors

Baseline characteristics, vascular risk factors, laboratory data, and medical documents were retrieved. Vascular risk factors, including hypertension (defined as a history of hypertension or diagnosed at discharge), diabetes (defined as a history of diabetes or diagnosed at discharge), hyperlipidemia (defined as a history of hyperlipidemia or received lipid-lowering treatments or diagnosed at discharge), history of myocardial infarction and smoking, were carefully identified according to our previous study (2).

### Neuroimaging Examinations

A brain MRI examination was performed in either a 3.0-T (Magentom Trio, Siemens, Erlangen, Germany) or 1.5-T (GE Medical Systems, Milwaukee, WI) system to obtain axial T1-weighted images, axial T2-weighted images, axial diffusion-weighted imaging (DWI), fluid-attenuated inversion recovery (FLAIR), 3D time-of-flight MRA and gradient-echo T2^*^-weighted or susceptibility-weighted imaging (SWI) images. All MRI source images were evaluated by two neurologists (CZ and LH) who were blinded to the clinical information.

### Intracranial Arterial Diameter Measurements and Brain Arterial Remodeling (BAR) Score

**Figure 1:**
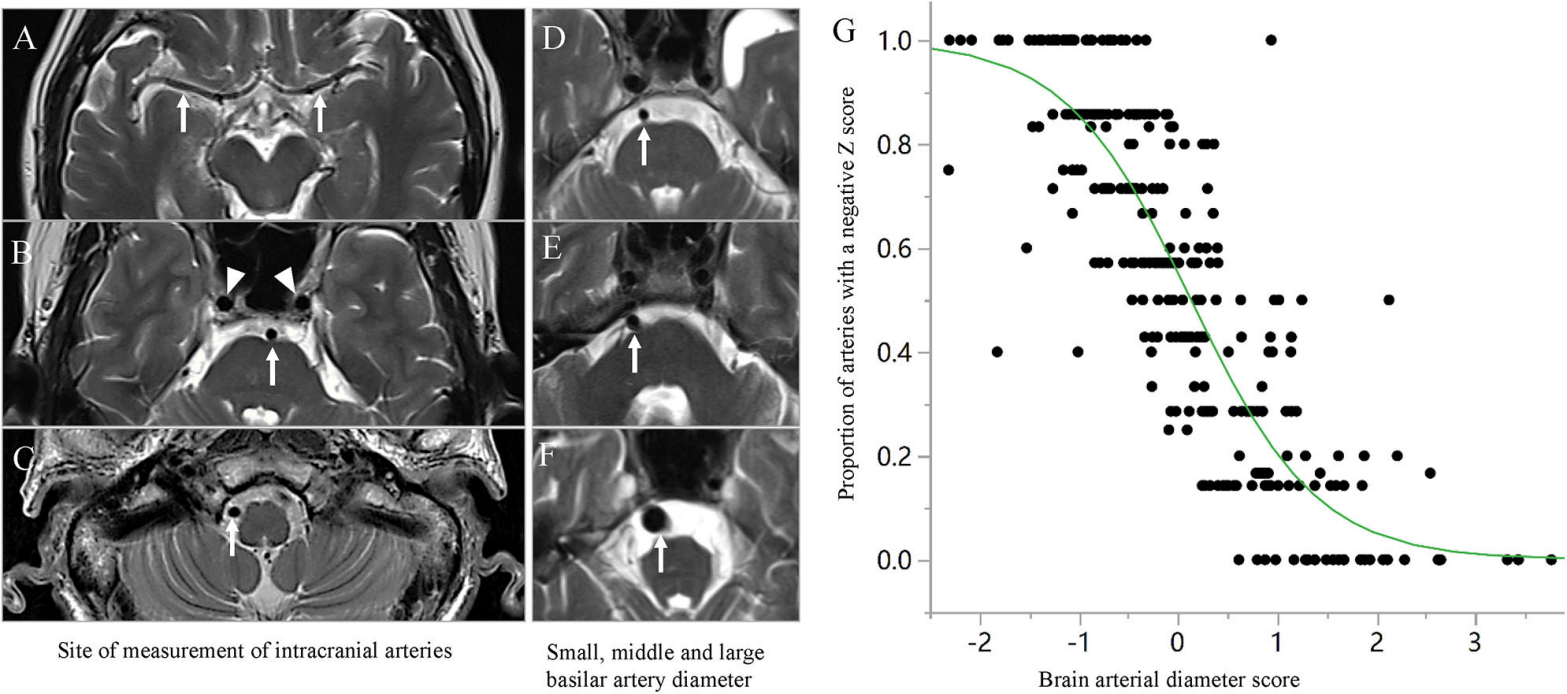
Spline regression fit for the proportion of arteries with a negative Z Score for lumen diameter. The diameters of main seven intracranial arteries were measured in the middle cerebral artery (R and L) at the M1 segment [**(A)**, arrow], the basilar artery at the mid-pons [**(B)**, arrow], the intracranial vertebral arteries (R and L) at the V4 segment [**(C)**, arrow], and the internal carotid arteries (R and L) at the intracavernous segment [**(B)**, arrowhead]. **(D–F)** show the small, middle, and large basilar arteries, respectively (arrow). The BAR score is a construct that discloses the tendency of an individual to have small or large arteries in a single number. As the score decreases, the proportion of arteries with small lumina increases and vice versa **(G)**.

Diameters of the main seven intracranial arteries (Figures 1A–C), including the internal carotid arteries (ICA) (R and L) at the intra-cavernous segment, the MCA (R and L) at the M1 segment, the basilar artery (BA) at the mid-pons (Figures 1D–F) and the intracranial vertebral arteries (VA) (R and L) at the V4 segment, were measured according to our previous study (2). An average arterial *Z*-score, also named the brain arterial remodeling (BAR) score, for each individual was obtained by adding all measured arteries and dividing by the total number of identified arteries as described in a previous study (1). The BAR score was normalized and then used both continuously and categorically, using three categories: (1) “individuals with large diameters” for participants with a BAR score>1 SD, (2) “individuals with small diameters” for participants with a BAR score < -1 SD, and (3) “individuals with average diameters” for participants with a BAR score between −1 and 1 SD. Within each individual, the greater the number of arteries with negative scores, the more likely it was that the BAR score had a negative overall *Z*-score and vice versa (Figure 1G).

### Definition of the Total CSVD Score

The total CSVD score including 4 MRI markers of CSVD (lacuna, WMH, EPVS, and CMBs) was defined according to previous studies (9, 10). Briefly, a lacuna was described as a round or ovoid hyperintense lesion on T2-weighted images, 3 and 15 mm in diameter, with a surrounding rim of hyperintensity on FLAIR but negative on DWI (11). WMH rated on FLAIR images was described using the modified Fazekas score (12), a periventricular WMH Fazekas score of 3 or a deep WMH Fazekas score of 2 or 3 was defined as severe WMH. EPVS in basal ganglia and centrum semiovale regions were rated. They were defined as small (<3 mm), punctate or linear-shaped lesions with a cerebrospinal fluid-like signal on all MRI sequences but without a hyperintense rim on T2-FLAIR (11). The number of EPVS was rated as follows: 0 to 10 EPVS (mild); 11 to 25 EPVS (moderate); and >25 EPVS (severe) in both anatomic areas. CMBs were defined on gradient-echo T2^*^ or SWI as small (<10 mm), homogenous, round, low-signal intensities (13). An ordinal total CSVD score ranging from 0 to 4 was calculated by counting the above 4 MRI features. One point was awarded for each of the following items: ≥1 asymptomatic lacuna (1 point if present); periventricular WMH Fazekas score 3 or if deep WMH Fazekas score 2 or 3 (1 point if present); moderate to severe EPVS in the basal ganglia (1 point if present); and any CMBs (1 point if present) (9). Intrarater reliability testing (50 scans) showed a good reliabilitywith the kappa values for the presence of lacuna of 0.77, basal ganglia EPVS of 0.80, centrum semiovale EPVS of 0.75, deep WMH of 0.77, periventricular WMH of 0.73 and the CMBs of 0.82.

### Statistical Analyses

Continuous variables are presented as the mean ± SD. Categorical variables were recorded as proportions. Between-group comparisons of the distribution of continuous variables were performed using a one-way ANOVA or independent samples *t*-test. Comparisons of categorical variables were performed using the χ^2^-test or Fisher's exact test. We first investigated whether the demographic and vascular risk factors varied across the 3 remodeling groups. We then assessed the relationship between the BAR score and all CSVD signs with binary logistic regression. The correlation between the BAR score and total CSVD score was measured with the Spearman's rank correlation coefficient method. The relationship between the BAR score and the total CSVD score was evaluated with ordinal logistic regression analysis. Age, sex, and risk factors with a *P*-value of <0.1 in the univariate analysis were included in multivariate analysis. All statistical testing was two-tailed, and *P* < 0.05 was considered statistically significant. All analyses were performed with IBM SPSS Statistics 25.0 (IBM, Armonk, NY).

## Results

### Sample Description

A total of 312 patients were involved in this study, with a mean age of 59.9 ± 11.1 years, and 73.4% were men. The mean body surface area was 1.45 ± 0.73 m^2^. A total of 203 (65.1%) patients had hypertension, 83 (26.6%) patients had diabetes mellitus, 27 (8.7%) had hyperlipidemia and 17 (5.4%) had a previous myocardial infarction. The mean diameters of the main seven intracranial arteries were as follows: BA, 3.4 ± 0.8 mm; RVA, 2.2 ± 0.8 mm; LVA, 2.8 ± 0.8 mm; RICA, 4.7 ± 0.7 mm; LICA, 4.7 ± 0.8 mm; RMCA, 2.4 ± 0.4 mm; and LMCA, 2.4 ± 0.5 mm. During hospitalization, 97.1% of patients received antiplatelet therapy, 13.1% of patients received anticoagulant therapy, and 89.1% of patients received statin therapy (Supplementary Table 1).

### Baseline Characteristics of the Participants According to BAR Category

Forty-five patients had small brain arterial diameters (BAR < −1 SD), 47 patients had large brain arterial diameters (BAR > 1 SD) and 220 patients had average diameters (−1 SD ≤ BAR ≤ 1 SD). Patients with large arterial diameters had older ages (*P* = 0.039) and more previous myocardial infarctions than other patients (*P* = 0.033). The hypertension rate was increased gradually according to the increase in diameter (51.1%, 63.6%, and 85.1% for BAR < -1 SD, −1 SD ≤ BAR ≤ 1 SD, and BAR >1 SD, respectively); however, it was not statistically significant (*P* = 0.647). We did not find a significant difference according to the gradual increase in intracranial arterial diameter concerning sex (*P* = 0.270), diabetes mellitus (*P* = 0.353), hyperlipidemia (*P* = 0.184), and smoking (*P* = 0.744, Table 1).

**Table 1:** Baseline characteristics of participants according to remodeling category.

**Characteristics**	**Brain arterial remodeling score**			***P*-value^†^**
	** < −1 SD *n* = 45**	**−1 to 1 SD *n* = 220**	**> 1 SD *n* = 47**	
Age, y	54.7 ± 11.5	59.9 ± 11.1	64.9 ± 8.8	0.039
Male	28 (62.2)	167 (75.9)	34 (72.3)	0.270
BSA, m^2^	1.42 ± 0.73	1.43 ± 0.74	1.55 ± 0.67	0.592
Hypertension	23 (51.1)	140 (63.6)	40 (85.1)	0.647
Diabetes mellitus	8 (17.8)	62 (28.2)	13 (27.7)	0.353
Myocardial infarction	1 (2.2)	10 (4.5)	6 (12.8)	0.033
Hyperlipidemia	7 (15.6)	17 (63.0)	3 (11.1)	0.184
Smoking	16 (36.4)	91 (41.6)	15 (31.9)	0.744
Asymptomatic large artery stenosis	4 (8.9)	21 (9.5)	6 (12.8)	0.785
Hct	0.408 ± 0.045	0.410 ± 0.043	0.407 ± 0.051	0.826
WBC, × 10^9^/L	6.90 ± 2.87	7.63 ± 3.41	6.98 ± 2.06	0.223
Ca^2+^, mmol/L	2.18 ± 0.12	2.16 ± 0.11	2.17 ± 0.09	0.663
TC, mmol/L	4.36 ± 1.25	4.24 ± 0.96	4.19 ± 1.11	0.413
LDL, mmol/L	2.55 ± 0.91	2.49 ± 0.82	2.31 ± 0.77	0.343
HDL, mmol/L	1.14 ± 0.46	1.03 ± 0.27	1.09 ± 0.29	0.296
HbA1c	6.9 ± 2.5	6.4 ± 1.4	6.6 ± 1.7	0.055

*Logistic regression proportional odds models were used to assess the strength of association*.

† *P value adjusted by age, sex, BSA, hypertension, diabetes mellitus, myocardial infarction, hyperlipidemia, smoking, and laboratory tests. BSA, body surface area; Ca^2+^, calcium; HbA1c, glycosylated hemoglobin; Hct, hematocrit; HDL, high-density lipoprotein cholesterol; LDL, low-density lipoprotein cholesterol; TC, total cholesterol; WBC, white blood cell*.

### Risk-Factors Related to CSVD and the Total CSVD Score (Univariate Analysis)

total of 201 patients had CSVD features, univariate analysis found that age (61.9 ± 10.4 vs. 56.3 ± 11.6 for patients with and without CSVD, respectively, *P* < 0.001), hypertension (78.1 vs. 41.4%, respectively, *P* < 0.001), diabetes mellitus (30.8 vs. 18.9%, respectively, *P* = 0.022) and BAR score (*P* = 0.001) were correlated with CSVD. Ordinal logistic regression analysis found that the total CSVD score was correlated with age (crude OR = 1.047; 95% CI, 1.027–1.067; *P* < 0.001); hypertension (crude OR = 4.989; 95% CI, 3.136–7.938; *P* < 0.001) and BAR score> 1 SD (crude OR = 3.311; 95% CI, 1.872–5.855; *P* < 0.001). However, it seemed that small arterial diameter was not correlated with total CSVD score (crude OR = 0.686; 95% CI, 0.380–1.240; *P* = 0.213, Table 2). The percentage of total CSVD score = 0 declined gradually with increasing BAR score (Supplementary Figure 1) and the Spearman's rank correlation coefficient between BAR score and total burden of CSVD was 0.320 (*P* < 0.001).

**Table 2:** Characteristics of the study population stratified by CSVD and the total CSVD score (Univariate analysis).

**Characteristics**	**Without CSVD *n* = 111**	**With CSVD *n* = 201**	***P*-value**	**Total CSVD score**					***P*-value**
				**0 (*n* = 111)**	**1 (*n* = 83)**	**2 (*n* = 59)**	**3 (*n* = 43)**	**4 (*n* = 16)**	
Age	56.3 ± 11.6	61.9 ± 10.4	<0.001	56.3 ± 11.6	60.3 ± 10.4	62.5 ± 9.9	63.3 ± 11.3	64.6 ± 9.4	<0.001
Male	85 (76.6)	144 (62.9)	0.345	85 (76.6)	61 (73.5)	43 (72.9)	27 (62.8)	13 (81.3)	0.283
Hypertension	46 (41.4)	157 (78.1)	<0.001	46 (41.4)	59 (71.1)	43 (72.9)	41 (95.3)	14 (87.5)	<0.001
Diabetes mellitus	21 (18.9)	62 (30.8)	0.022	21 (18.9)	19 (22.9)	20 (33.9)	17 (39.5)	6 (37.5)	0.002
MI	3 (2.7)	14 (7.0)	0.112	3 (2.7)	6 (7.2)	3 (5.1)	3 (7.0)	2 (12.5)	0.137
Hyperlipidemia	6 (5.4)	21 (10.4)	0.129	6 (5.4)	7 (8.4)	6 (10.2)	7 (16.3)	1 (6.3)	0.088
Smoke	50 (45.5)	72 (36.0)	0.103	50 (45.5)	32 (39.0)	20 (33.9)	13 (30.2)	7 (43.8)	0.092
BAR score^†^			0.001						
−1 to 1 SD	84 (75.7)_a_	136 (67.7)_a_		84 (75.7)	59 (71.1)	41 (69.5)	27 (62.8)	9 (56.3)	–
<−1 SD	21 (18.9)_a_	24 (11.9)_a_		21 (18.9)	12 (14.5)	7 (11.9)	4 (9.3)	1 (6.3)	0.213
>1 SD	6 (5.4)_a_	41 (20.4)_b_		6 (5.4)	12 (14.5)	11 (18.6)	12 (27.9)	6 (37.5)	<0.001
Antiplatelet	106(95.5)	197 (98.0)	0.359	106 (95.5)	81 (97.6)	57 (96.6)	43 (100)	16 (100)	0.168
Anticoagulant	17 (15.3)	24 (11.9)	0.398	17 (15.3)	14 (16.9)	7 (11.9)	3 (7.0)	0 (0.0)	0.096
Statin	96 (86.5)	182 (90.5)	0.270	96 (86.5)	72 (86.7)	55 (93.2)	40 (93.0)	15 (93.8)	0.117

*BAR, brain arterial remodeling; MI, myocardial infarction*.

† *Each subscript letter (_a_) denotes a subset of CSVD categories whose column proportions do not differ significantly from each other at the 0.05 level*.

### BAR Score and Its Relationship With Separate CSVD Signs

After adjusting for age and sex, BAR score >1 SD was correlated with lacuna (OR = 2.163; 95% CI, 1.138–4.111; *P* = 0.019), severe WMH (OR = 2.221; 95% CI, 1.128–4.375; *P* = 0.021), and severe EPVS (OR = 2.782; 95% CI, 1.435–5.394; *P* = 0.002); BAR score >1 SD was not correlated with CMBs (OR = 1.171; 95% CI, 0.881–3.282; *P* = 0.113); and patients with BAR score > 1 SD were more prone to developing CSVD (OR=3.625; 95% CI, 1.446–8.967; *P* = 0.005, Table 3). After adjusted for age, sex, hypertension, diabetes mellitus, BAR score >1 SD remained correlated with lacuna (OR = 1.987; 95% CI, 1.037–3.807; *P* = 0.039), severe WMH (OR = 1.994; 95% CI, 1.003–3.964; *P* = 0.049), severe EPVS (OR = 2.544; 95% CI, 1.299–4.983; *P* = 0.006) and CSVD (OR = 2.997; 95% CI 1.182–7.599; *P* = 0.021, Table 3).

**Table 3:** BAR score in relation to cerebral small vessel signs (binary logistic regression).

	**Lacuna OR (95% CI)**	**Severe WMH OR (95% CI)**	**Severe EPVS OR (95% CI)**	**CMBs OR (95% CI)**	**CSVD OR (95% CI)**
Model 1					
BAR score> 1 SD	2.163 (1.138–4.111)*	2.221(1.128–4.375)*	2.782 (1.435–5.394)^†^	1.171 (0.881–3.282)	3.625 (1.466–8.967)^†^
Model 2					
BAR score> 1 SD	1.987 (1.037–3.807)*	1.994 (1.003–3.964)*	2.544 (1.299–4.983)^†^	1.410 (0.702–2.832)	2.997 (1.182–7.599)*

*BAR, brain arterial remodeling; CMBs, cerebral microbleeds; CSVD, cerebral small vessel disease; EPVS, enlarged perivascular spaces; IADE, intracranial arterial dolichoectasia; WMH, white matter hyperintensity*.

*Model 1, adjusted for age and sex; Model 2 adjusted for age, sex, hypertension, diabetic mellitus*.

* *P < 0.05, the detailed P-value was recorded in the manuscript*.

† *P < 0.01, the detailed P-value was recorded in the manuscript*.

### Associations With Total CSVD Score in the Multivariable Ordinal Regression Analysis

We first analyzed the BAR score as dichotomous independent variables (>1 SD vs. ≤ 1 SD), and multivariable ordinal logistic regression detected that age (OR = 1.028; 95% CI, 1.008–1.049; *P* = 0.006), hypertension (OR = 3.529; 95% CI, 2.152–5.787; *P* < 0.001), and BAR score >1 SD (OR = 2.457; 95% CI, 1.379–4.376; *P* < 0.001) were correlated with an increase in total CSVD score. We then analyzed the BAR score by using the participants with average diameters (−1 to 1 SD) as the reference group. Multivariable ordinal logistic regression also found that age (OR = 1.028; 95% CI, 1.007–1.049; *P* = 0.009), hypertension (OR = 3.514; 95% CI, 2.114–5.769; *P* < 0.001), BAR score >1SD (OR = 2.418; 95% CI, 1.350–4.330; *P* = 0.003) were correlated with the total CSVD score. We did not find that BAR score < − 1 SD correlated with the total CSVD score (OR = 0.880; 95% CI, 0.461–1.678; *P* = 0.695, Table 4).

**Table 4:** Associations with total CSVD score in multivariable ordinal logistic regression analysis.

**Characteristics**	**OR (95% CI)**	***P*-value**
Model 1		
Age	1.028 (1.008–1.049)	0.006
Male	1.004 (0.588–1.713)	0.988
Hypertension	3.529 (2.152–5.787)	<0.001
Diabetes mellitus	1.497 (0.933–2.401)	0.093
Hyperlipidemia	1.736 (0.818–3.681)	0.151
Smoking	0.953 (0.585–1.522)	0.848
BAR score > 1 SD	2.457 (1.379–4.376)	0.002
Anticoagulant	0.580 (0.311–1.080)	0.091
Model 2		
Age	1.028 (1.007–1.049)	0.009
Male	0.990 (0.577–1.697)	0.970
Hypertension	3.514 (2.144–5.769)	<0.001
Diabetes mellitus	1.486 (0.925–2.387)	0.099
Hyperlipidemia	1.756 (0.825–3.738)	0.145
Smoking	0.951 (0.584–1.550)	0.842
BAR score		
−1 to 1 SD	Reference	–
< −1 SD	0.880 (0.461–1.678)	0.695
>1 SD	2.418 (1.350–4.330)	0.003
Anticoagulant	0.655 (0.351–1.221)	0.091

*In Model 1, the BAR score was analyzed as dichotomous independent variables (>1SD vs. ≤1SD). In model 2, the BAR score was analyzed by using the participants with average diameters (−1 to 1 SD) as the referent group. BAR, brain arterial remodeling*.

## Discussion

A previous study has suggested that the incidence rates for death, vascular death, myocardial infarction, and any vascular event were higher in individuals with the largest arterial diameters (1). The hemodynamic burden may be transmitted through an intracranial large artery to the downstream small vessels (3) and then cause small vessel disease. In this study, we provided evidence that large brain artery caliber is associated with the total CSVD score, as well as lacuna, WMH, and EPVS.

Our study found that large arterial diameter was correlated with age and myocardial infarction, but not with other atherosclerosis risk factors, such as diabetes, smoking, and hyperlipidemia. Previous studies also found that extreme intracranial arterial outward remodeling, called dolichoectasia, was also correlated with myocardial infarction (14), coronary arterial ectasia (15), and an enlarged descending thoracic aorta (16). This finding may indicate that brain arterial outward remodeling may be a biomarker of systemic arterial stiffness (17).

CSVD is a dynamic, whole-brain disorder and a common cause of dementia, stroke, and gait disturbances. Previous studies suggested a strong relationship between large intracranial arterial diameter and lacuna infarction (4), WMH (18), EPVS (5), and CMBs (7). However, previous studies only studied one or two large brain arteries, and selected separated CSVD indicators. It has been widely accepted that the total CSVD score is a more complete overall gauge of the impact of CSVD on the brain than are the individual MRI features separately. Our study found that large intracranial arterial diameter, as evaluated by the BAR score, was correlated with total CSVD score, which has seldom been reported to our knowledge. These findings suggested that the large intracranial arteries would be less able to dampen pressure and pulsatility, leading to more pulsatile energy dissipation in the brain and end-organ tissue damage (19).

A previous study found a positive correlation between large intracranial artiries outward remodeling and the severity of MRI markers of small vessel disease (18). which implies that the long-term hemodynamic burden caused by vascular wall remodeling may play an important role in the development of both large arteriopathy and small vessel disease.

Our study has some limitations. First, we did not calculate cerebral vessel pulsatility, as previous studies have shown that high pulsatility in the ICA or MCA is associated with WMH (3). Cerebral veins and CSF are also thought to be important compartments for compensating arterial pulse pressure (20). Second, we did not evaluate the MMP level, as a previous study suggested that MMP dysfunction could bridge large intracranial arterial outward remodeling and CSVD (21). Third, the measurement of intracranial artery calibers is affected by cardiac cycle and artery pulse and it might depend on the imaging time on MRA. For example, the average distension of the MCA area from diastole to systole was 2.58%. However, the phase-contrast flow velocity profiles found no significant correlation between MCA distension and the pulsatility index (22). Four, we do not analyze the brain arterial wall by HRMRI, a fact that weakens our claim that the diameters phenotypes represent remodeling phenotypes.

In summary, intracranial artery caliber is a biomarker for CSVD, and individuals with large arterial diameters have a greater risk of CSVD. The relationship between cerebral blood flow, cerebral vessel pulsatility, and CSVD needs further study.

## Data Availability Statement

The raw data supporting the conclusions of this article will be made available by the authors, without undue reservation.

## Ethics Statement

The studies involving human participants were reviewed and approved by the ethics committee of Jinling Hospital. The patients/participants provided their written informed consent to participate in this study.

## Author Contributions

ZC and GX: study design, interpretation of results and manuscript drafting. MW and CC: study design and interpretation of results. HL: data collection. XF: study design and statistical analysis. XL and GX: study design, statistical analysis and critical revision of manuscript. GX and ZC: have full access to all of the data in the study and take responsibility for the integrity of the data and the accuracy of the data analysis. All authors: contributed to the article and approved the submitted version.

## Conflict of Interest

The authors declare that the research was conducted in the absence of any commercial or financial relationships that could be construed as a potential conflict of interest.

## Supplementary Material

Supplementary Figure S1 Total CSVD burden according to the brain arterial remodeling score. The percentage of total CSVD score = 0 declined gradually with the increasing BAR score. The Spearman's rank correlation coefficient between the BAR score and total burden of CSVD is 0.320 (*P* < 0.001).

Supplementary Table S1 Baseline characteristics of the study population.
