# Supplementary figures and images for: Caliber of Intracranial Arteries as a Marker for Cerebral Small Vessel Disease
Source: Front Neurol. 2020 Sep 24;11:558858. doi: 10.3389/fneur.2020.558858 (PMC7542665; doi:10.3389/fneur.2020.558858)

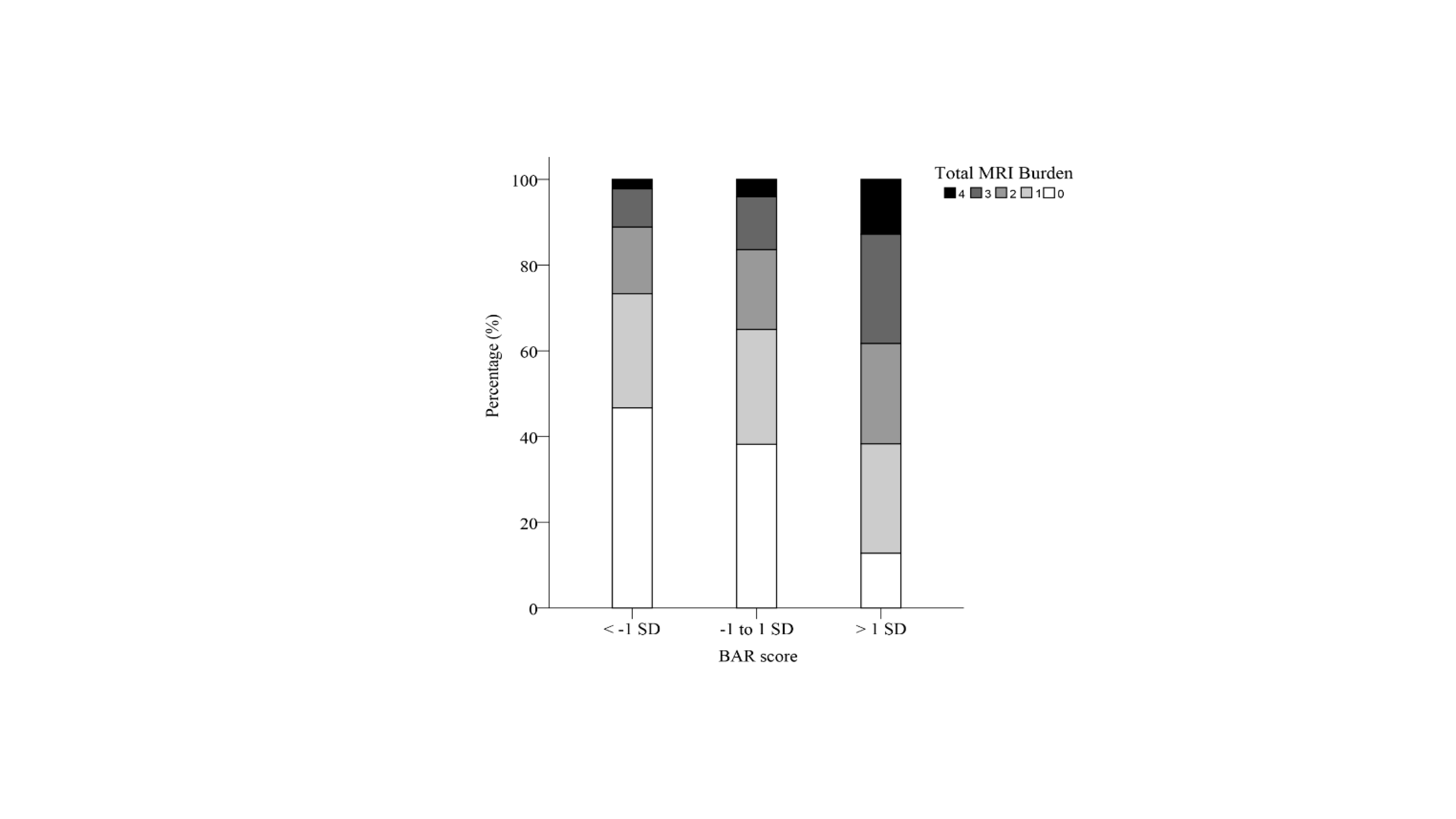

Supplement: Supplementary Figure S1 — Total CSVD burden according to the brain arterial remodeling score. The percentage of total CSVD score = 0 declined gradually with the increasing BAR score. The Spearman's rank correlation coefficient between the BAR score and total burden of CSVD is 0.320 (P < 0.001). [file Image_1.TIF]
