# Supplementary material for: Caliber of Intracranial Arteries as a Marker for Cerebral Small Vessel Disease
Source: Front Neurol. 2020 Sep 24;11:558858. doi: 10.3389/fneur.2020.558858 (PMC7542665; doi:10.3389/fneur.2020.558858)
Supplement: Supplementary Table S1 — Baseline characteristics of the study population. [file Table_1.DOCX]

**Supplemental Table S1** Baseline Characteristics of The Study Population

| Characteristics | Value (N = 312) |
| --- | --- |
| Age, y, mean ± SD | 59.9 ± 11.1 |
| Male, no./total no. (%) | 229/312 (73.4) |
| BMI, mean ± SD | 24.9 ± 3.1 |
| Body surface area, m^2^, mean ± SD | 1.45 ± 0.73 |
| Medical history, no./total no. (%) |  |
| Hypertension | 203/312 (65.1) |
| Diabetes mellitus | 83/312 (26.6) |
| Myocardial infarction | 17/312 (5.4) |
| Smoking | 122/310 (39.4) |
| Hyperlipidemia | 27/312 (8.7) |
| Intracranial Arterial Diameter^*^, mean ± SD |  |
| Basilar artery | 3.4 ± 0.8 |
| Right vertebral artery | 2.2 ± 0.8 |
| Left vertebral artery | 2.8 ± 0.8 |
| Right internal carotid artery | 4.7 ± 0.7 |
| Left internal carotid artery | 4.7 ± 0.8 |
| Right middle cerebral artery | 2.4 ± 0.4 |
| Left middle cerebral artery | 2.4 ± 0.5 |
| Blood pressure, mmHg, mean ± SD |  |
| Systolic | 143 ± 19 |
| Diastolic | 82 ± 12 |
| Laboratory tests |  |
| Hct | 0.409 ± 0.045 |
| WBC, ×10^9^/L, mean ± SD | 7.43 ± 3.17 |
| Ca^2+^, mmol/L, mean ± SD | 2.17 ± 0.11 |
| TC, mmol/L, mean ± SD | 4.25 ± 1.05 |
| LDL, mmol/L, mean ± SD | 2.47 ± 0.84 |
| HDL, mmol/L, mean ± SD | 1.06 ± 0.32 |
| HbA1c, mean ± SD | 6.5 ± 1.6 |
| In-hospital treatment |  |
| Antiplatelet | 303/312 (97.1) |
| Anticoagulant | 41/312 (13.1) |
| Statin | 278/312 (89.1) |

^*^Number calculated for intracranial artery diameter: basilar artery, n = 304; right vertebral artery, n = 287; left vertebral artery, n = 290; right internal carotid artery, n = 286; left internal carotid artery, n = 292; right middle cerebral artery, n = 260; left middle cerebral artery, n = 254. BMI, body mass index; Ca^2+^, calcium; HbA1c, glycosylated hemoglobin; Hct, hematocrit; HDL, high-density lipoprotein cholesterol; LDL, low-density lipoprotein cholesterol; TC, total cholesterol; WBC, white blood cell.
